# Non-Atriotomy Surgical Ablation Is Associated With a Reduction of Postoperative Atrial Fibrillation

**DOI:** 10.1016/j.atssr.2023.09.007

**Published:** 2023-09-27

**Authors:** Armin Kiankhooy, Federico Sertic, Michaela Daw, Susan Eisenberg, Arash Kiankhooy, Gansevoort Dunnington

**Affiliations:** 1 Department of Cardiothoracic Surgery, Smidt Heart Institute, Cedars-Sinai Medical Center, Los Angeles, California; 2 Department of Cardiothoracic Surgery, Adventist Health–St Helena Hospital, St Helena, California; 3 Department of Electrophysiology, Adventist Health–St Helena Hospital, St Helena, California; 4 George Washington University School of Medicine, Washington, DC

## Abstract

**Background:**

Postoperative atrial fibrillation (POAF) is common after cardiac operations, and effective intraoperative techniques aimed at reducing POAF focus on limiting left atrial triggers through posterior pericardiotomy or pulmonary vein isolation. Prophylactic left atrial appendage occlusion (LAAO) is increasingly used in hopes of preventing POAF-associated strokes. We sought to compare the incidence of POAF in patients undergoing prophylactic LAAO with or without a prophylactic non-atriotomy surgical ablation (NASA).

**Methods:**

A retrospective observational cohort comparison study of patients undergoing first-time isolated coronary artery bypass grafting (CABG) with LAAO only (n = 90) or NASA+LAAO (n = 42) was conducted from July 2020 through November 2022. In-hospital POAF was defined by standard Society of Thoracic Surgeons (STS) definitions using 24-hour continuous telemetry and daily electrograms. Standard STS outcomes were also examined. Data are represented as mean ± SD. *P* values <.05 are considered significant.

**Results:**

STS-collected patient demographics, operative characteristics, and major complications did not differ significantly between cohorts. The rate of POAF (LAAO only, 41.1%; NASA+LAAO, 4.7%; *P* < .0001; odds ratio, 0.07; 95% CI, 0.016-0.27) and amiodarone on discharge (LAAO only, 42.5%; NASA+LAAO, 7.3%; *P* < .0001; odds ratio, 0.11; 95% CI, 0.03-0.33) differed significantly between cohorts. Total hospital costs were similar.

**Conclusions:**

In patients with isolated coronary artery bypass grafting undergoing LAAO, a NASA is associated with a reduction of in-hospital POAF and need for antiarrhythmic medications on discharge. Future randomized prospective controlled studies are needed to prove safety and effectiveness.

## Visual Abstract

**Figure:**
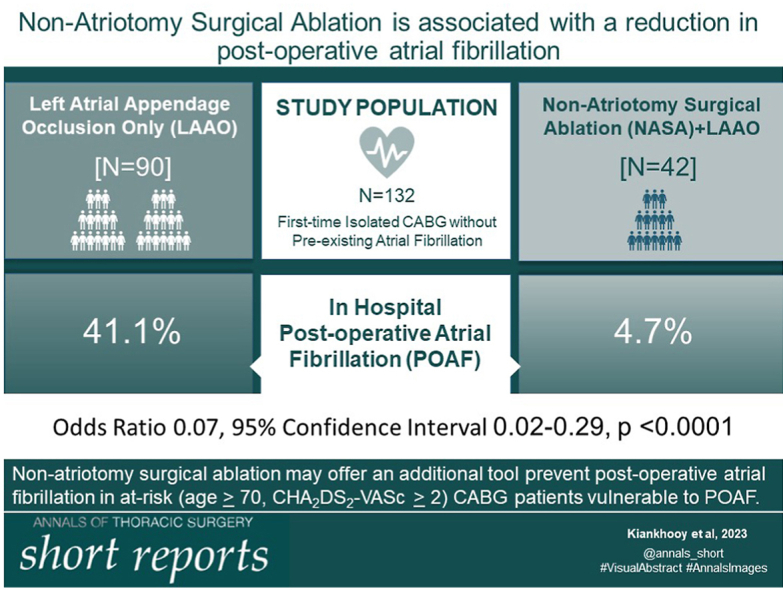

**In Short**

▪ Intraoperative techniques aimed at left atrial trigger reduction (posterior pericardiotomy and pulmonary vein isolation) lead to reductions of postoperative atrial fibrillation.
▪ A non-atriotomy surgical ablation of the left atrial posterior wall is associated with a reduction of postoperative atrial fibrillation in high-risk patients undergoing coronary artery bypass grafting.

Postoperative atrial fibrillation (POAF) is more than just an inconvenience; in fact, in patients undergoing coronary artery bypass grafting (CABG), POAF is linked to increased risk of stroke and death.^1^ Increasingly, cardiac surgeons are prophylactically managing the left atrial appendage in patients without preexisting atrial fibrillation in hopes of minimizing the risk of embolic stroke in the 20% to 50% of cardiac surgery patients who go on to have POAF.^2^ We routinely manage the left atrial appendage with an epicardial occlusion device in patients with increased risk for development of POAF (age, ≥70 years; CHA_2_DS_2_VASc score, ≥2). Lednev and colleagues^3^ in 2017 and more recently Willekes and coworkers^4^ examined the safety and efficacy of prophylactic bilateral pulmonary vein isolation to reduce POAF in the setting of left atrial appendage amputation. The rate of POAF in this primarily isolated CABG (50/60 [83%]) population was significantly less in the treatment cohort than in the control cohort (7% vs 55%; *P* < .001). This poses an attractive approach of POAF reduction by prophylactic electrical isolation while maintaining stroke mitigation with prophylactic left atrial appendage management and possibly providing an all-encompassing optimal protective strategy for high-risk patients. Yates and associates^5^ newly reported the improved ability of a novel radiofrequency clamp to provide a non-atriotomy surgical ablation (NASA) with complete posterior wall isolation in comparison to the traditional radiofrequency bipolar clamp used by Willekes and coworkers (Figure 1). Therefore, we sought to observe the incidence of in-hospital POAF in an isolated CABG population undergoing routine left atrial appendage occlusion (LAAO) with an epicardial occlusion device with or without the addition of a prophylactic NASA.

**Figure 1:**
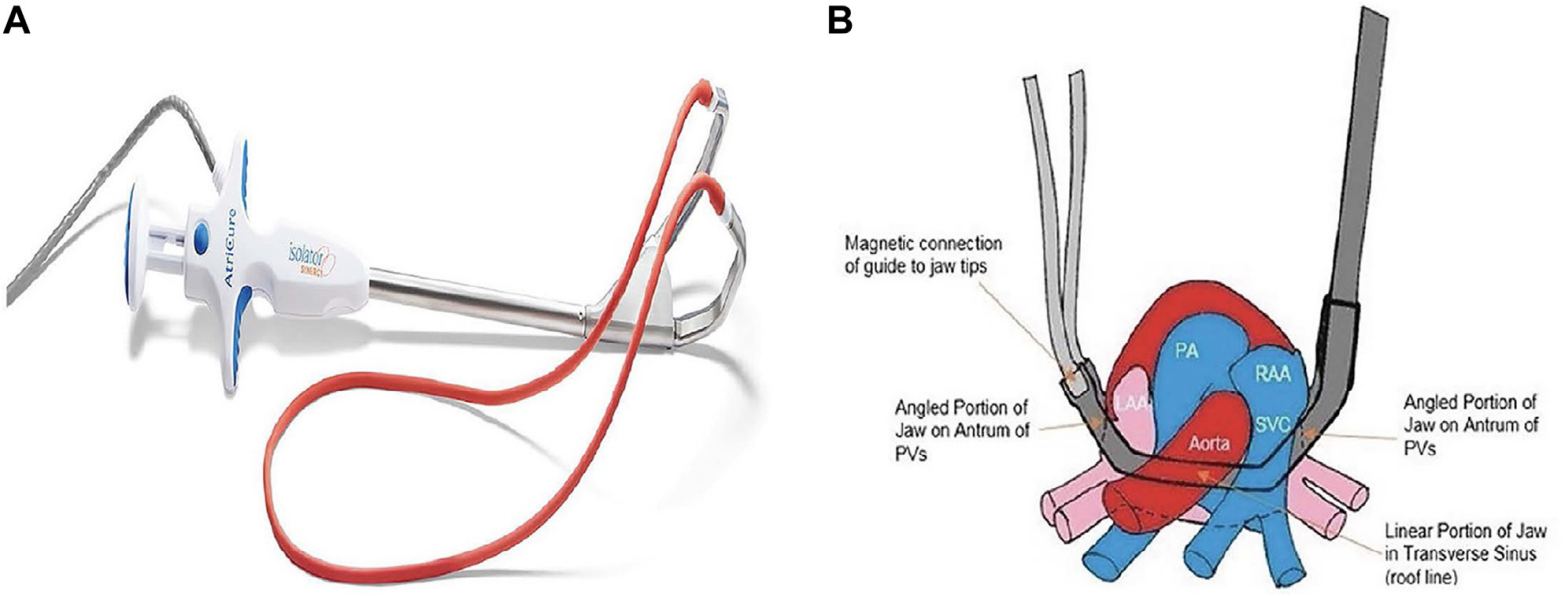
(A) The EnCompass device (AtriCure) is a nonirrigated dual-electrode bipolar radiofrequency clamp. The magnetized red rubber guide system is used to assist in the placement of the clamp. (B) The EnCompass clamp creates a box lesion that isolates the entire left atrial posterior wall in a single application. (LAA, left atrial appendage; PA pulmonary artery; PVs, pulmonary veins; RAA, right atrial appendage; SVC, superior vena cava.) Reprinted from Yates and colleagues^5^ with permission.

## Patient and Methods

### Study Design and Population

The Adventist Health–St Helena Hospital institutional review board approval was obtained to analyze this patient cohort, and further individual patient consent was waived owing to the retrospective nature of the study (approved February 7, 2023; IRB# not assigned). At the time of the index cardiac surgical consent, the risks and benefits of LAAO only or NASA+LAAO were discussed with the patients.

**Figure 2:**
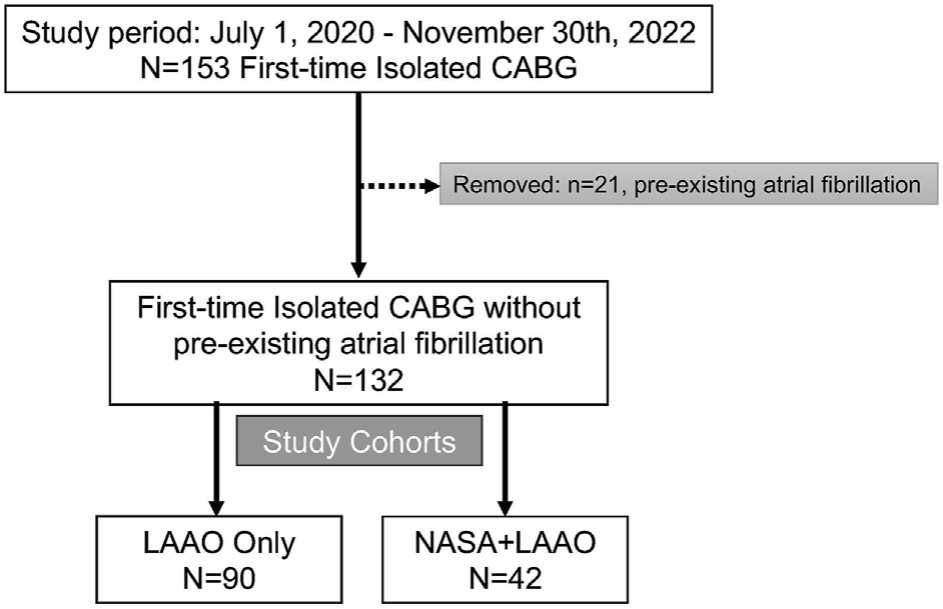
Patient flow diagram. Total study population, 132 patients. The comparison cohort included only patients undergoing first-time isolated coronary artery bypass grafting (CABG) without preexisting atrial fibrillation; 90 patients with left atrial appendage occlusion (LAAO) only, and 42 patients with non-atriotomy surgical ablation (NASA) + LAAO.

We performed a retrospective review of all consecutive cardiac operation patients treated at a single institution from July 1, 2020, until November 30, 2022. The review identified 153 patients undergoing first-time isolated CABG, refined to 132 patients without preexisting atrial fibrillation. The final 2 comparison cohorts included 90 patients with LAAO only and 42 patients with NASA+LAAO (Figure 2).

### Rhythm Monitoring

Patients were monitored for POAF during their index hospitalization with standard continuous 24-hour telemetry monitoring and daily 12-lead electrograms. POAF was defined by The Society of Thoracic Surgeons (SEQ#6496) definition of whether the patient experienced atrial fibrillation or flutter after operating room exit that lasts longer than 1 hour or lasts less than 1 hour but requires medical or procedural intervention. Standard clinical outcomes data were retrieved by review of the electronic medical record and our center-specific Society of Thoracic Surgeons–collected data.

### NASA and LAAO

The Isolator Synergy EnCompass device (AtriCure) is a 510(k) approved dual-electrode bipolar radiofrequency nonirrigated clamp with a 105-mm electrode length for cardiac tissue ablation (Figure 1). The clamp is positioned around the posterior left atrium through the oblique and transverse cardiac sinuses with a magnet-tipped red rubber catheter loop. This maneuver was performed with patients on cardiopulmonary bypass but without cardiac arrest. Five total transmural ablations were performed to isolate the posterior left atrium. Acute electrical isolation was confirmed with pace-capture testing at 20 mA at each pulmonary vein. The left atrial appendage epicardial occlusion was accomplished by application of an epicardial clip (AtriClip) as previously described.^6^

### Statistics

Statistical analysis was performed with Prism 9.2.0 (GraphPad Software). Variables are reported as mean with SD unless otherwise noted. Differences in continuous variables were analyzed by the unpaired nonparametric Mann-Whitney comparative ranks test. Differences in proportions of categorical variables were determined by the Fisher exact test. All *P* values are 2 sided with significance defined as <.05.

## Results

Patient demographics were not statistically different between the 2 groups (Table). Baseline left ventricular ejection fraction, total number of bypass grafts performed, and cardiopulmonary bypass and aortic cross-clamp times were not significantly different. However, on average, NASA+LAAO required 12 minutes of additional cardiopulmonary bypass time compared with LAAO only (107 ± 44 minutes vs 95 ± 23 minutes). No NASA-related procedural complications occurred.

**Table:** Patient Demographics and Outcomes

Parameter	LAAO Only (n = 90)	NASA+LAAO (n = 42)
Sex, female	21 (23.3)	7 (16.7)
Age ≥70 years	34 (37.8)	16 (38.1)
Age, y	66 ± 9.6	67 ± 10
BMI, kg/m^2^	30 ± 6	28 ± 5
CHA_2_DS_2_-VASc score	3.0 ± 1.2	3.2 ± 1.1
LVEF, %	50 ± 10	48 ± 11
Left atrial diameter, cm	4.0 ± 0.5	3.9 ± 0.6
Total No. of bypass grafts	3.2 ± 0.8	3.0 ± 0.6
Bypass duration, min	95 ± 23	107 ± 44
Cross-clamp duration, min	74 ± 19	75 ± 30
Length of stay, surgery to discharge	7.2 ± 4.1	7.6 ± 4.3
Beta blockers	72 (84.7)	36 (87.8)
Any blood product use	23 (25.6)	15 (35.7)
Renal failure requiring dialysis	2 (2.2)	2 (4.8)
Stroke	3 (3.3)	0 (0)
Death within 30 days	3 (3.3)	1 (2.4)
Total hospitalization cost, $	89,151 ± 38,475	86,674 ± 22,379
Total direct costs, $	51,217 ± 29,561	49,641 ± 13,725

Categorical variables are presented as number (percentage). Continuous variables are presented as mean ± SD.

BMI, body mass index; CHA_2_DS_2_-VASc, congestive heart failure, hypertension, age ≥75 years, diabetes, stroke, vascular disease, age 65 to 74 years, sex category (female); LAAO, left atrial appendage occlusion; LVEF, left ventricular ejection fraction; NASA, non-atriotomy surgical ablation.

### Rhythm Outcomes

**Figure 3:**
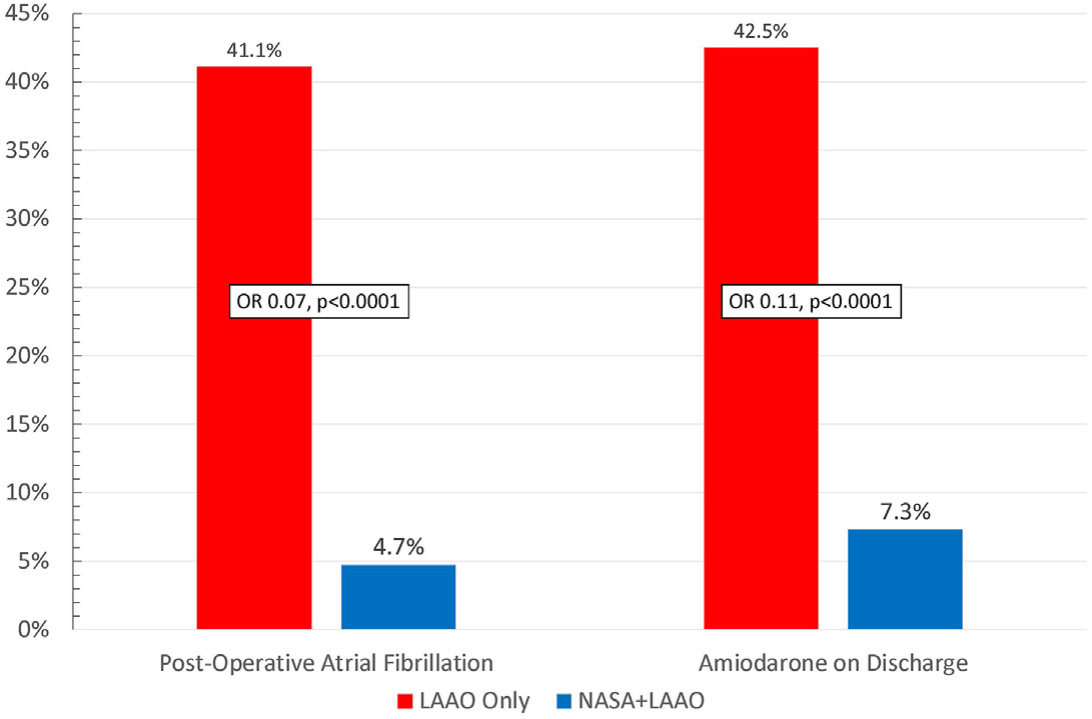
Rhythm-related patient outcomes. Patients undergoing left atrial appendage occlusion (LAAO) only (n = 90) suffered from significantly increased rates of postoperative atrial fibrillation and use of amiodarone on hospital discharge compared with non-atriotomy surgical ablation (NASA) + LAAO (n = 42). (OR, odds ratio.)

Patients who underwent LAAO only experienced a significantly greater incidence of POAF (LAAO only, 41.1%; NASA+LAAO, 4.7%; odds ratio, 0.07; 95% CI, 0.02-0.29; *P* <.0001) and amiodarone use on discharge (LAAO only, 42.5%; NASA+LAAO, 7.3%; odds ratio, 0.11; 95% CI, 0.03-0.33; *P* < .0001; Figure 3).

### Stroke

Three (3.3%) strokes occurred in the LAAO only cohort, whereas no strokes occurred in the NASA+LAAO cohort. One stroke occurred in a patient in whom POAF developed and who experienced postoperative mild dysarthria with a small lacunar stroke; the patient recovered without incident. One patient exhibited immediate global neurologic deficits postoperatively, and brain imaging revealed multiple areas of infarction; this patient was transitioned to comfort measures and died. The third stroke occurred in a patient who required cardiopulmonary resuscitation in the interventional suite before emergent CABG and exhibited a large left posterior cerebral infarction; this patient was also transitioned to comfort measures and died postoperatively.

### Deaths

One death occurred in a patient who postoperatively left against medical advice and was readmitted in severe heart and respiratory failure; despite maximal efforts, this patient could not be rescued and died. One death on postoperative day 5 was due to an acute hypoxic respiratory arrest.

### Hospital Costs

Although neither was statistically different, total hospitalization cost (LAAO only, $89,151 ± $38,475; NASA+LAAO, $86,674 ± $22,379) and direct costs (LAAO only, $51,217 ± $29,561; NASA+LAAO $49,641 ± $13,725) were less in the NASA+LAAO (Table).

## Comment

In this retrospective observational cohort comparison study, patients undergoing isolated CABG who received a prophylactic NASA and LAAO with an epicardial device demonstrated significantly less POAF (4.7%) than patients who received LAAO only (41.1%).

The recent prospective randomized controlled PALACS trial (posterior left pericardiotomy for the prevention of atrial fibrillation after cardiac surgery) demonstrated that a simple drainage procedure to allow egress of posterior pericardial fluid away from the posterior left atrium can lead to a significant reduction in POAF.^7^ However, notably, this technique was less effective in patients older than 70 years, patients with depressed left ventricular ejection fraction (<50%), or patients undergoing CABG. This may suggest that in patients with these attributes, a simple drainage procedure is insufficient and POAF prevention may require substrate modification of an underlying left atrial myopathy. In comparison, our study cohort comprised only CABG patients, nearly 40% of whom were older than 70 years and on average had a ventricular ejection fraction <50%. Despite these seemingly high-risk traits, prophylactic NASA was associated with minimal POAF (4.7%).

Willekes and coworkers specifically also addressed this population of higher risk patients. In their study, patients undergoing substrate modification with bilateral pulmonary vein isolation experienced significantly less POAF (7%) compared with the control group (55%). A common critique of this study is the high control group incidence of POAF (55%). In the prospective randomized ATLAS study (prophylactic left atrial appendage exclusion in cardiac surgery patients with elevated CHA_2_DS_2_-VASc score), Gerdisch and coworkers^2^ also reported evidence that prophylactic left atrial appendage management indeed is associated with an increased incidence of POAF (47.3% vs 38.2%; *P* = .047), albeit without an increased incidence of stroke (1.7% vs 2.8%; *P* = NS). We also reveal a high POAF rate in the LAAO only cohort (41.1%), and surgeons need to be aware of this expected increased rate with prophylactic LAAO.

Finally, hospital resource utilization and cost are important factors in POAF treatment and prevention. Despite the inherent increased direct cost of the EnCompass clamp used for NASA, the overall hospital direct costs and total hospitalization costs were similar in the 2 study cohorts. This may suggest that prevention of POAF ultimately may be cost-neutral while simultaneously being clinically beneficial. This could be a highly effective protective strategy to prevent POAF and POAF-related embolic stroke in high-risk patients who are less likely to respond to a left posterior pericardiotomy.

### Limitations

Because of the retrospective nature of this study, associations of NASA+LAAO and LAAO only treatments with POAF and additional clinical outcomes can only be proposed. No causative relationships are established in this preliminary study. The study is also underpowered to identify differences in rare events (eg, stroke), and so these comparisons should be interpreted with caution.

### Conclusion

In a retrospective observational cohort comparison study, a prophylactic NASA with LAAO was associated with significantly less POAF and reduced use of amiodarone on discharge in an isolated CABG population.
